# Biotic Interactions in Microbial Communities as Modulators of Biogeochemical Processes: Methanotrophy as a Model System

**DOI:** 10.3389/fmicb.2016.01285

**Published:** 2016-08-23

**Authors:** Adrian Ho, Roey Angel, Annelies J. Veraart, Anne Daebeler, Zhongjun Jia, Sang Yoon Kim, Frederiek-Maarten Kerckhof, Nico Boon, Paul L. E. Bodelier

**Affiliations:** ^1^Department of Microbial Ecology, Netherlands Institute of Ecology (NIOO-KNAW)Wageningen, Netherlands; ^2^Department of Microbiology and Ecosystem Science, Division of Microbial Ecology, Research Network Chemistry meets Microbiology, University of ViennaVienna, Austria; ^3^State Key Laboratory of Soil and Sustainable Agriculture, Institute of Soil Science, Chinese Academy of SciencesNanjing, China; ^4^Center for Microbial Ecology and Technology, Faculty of Bioscience Engineering, Ghent UniversityGhent, Belgium

**Keywords:** microbial interaction, microbial network, methanotrophy, methane oxidation, ecosystem functioning

## Abstract

Microbial interaction is an integral component of microbial ecology studies, yet the role, extent, and relevance of microbial interaction in community functioning remains unclear, particularly in the context of global biogeochemical cycles. While many studies have shed light on the physico-chemical cues affecting specific processes, (micro)biotic controls and interactions potentially steering microbial communities leading to altered functioning are less known. Yet, recent accumulating evidence suggests that the concerted actions of a community can be significantly different from the combined effects of individual microorganisms, giving rise to emergent properties. Here, we exemplify the importance of microbial interaction for ecosystem processes by analysis of a reasonably well-understood microbial guild, namely, aerobic methane-oxidizing bacteria (MOB). We reviewed the literature which provided compelling evidence for the relevance of microbial interaction in modulating methane oxidation. Support for microbial associations within methane-fed communities is sought by a re-analysis of literature data derived from stable isotope probing studies of various complex environmental settings. Putative positive interactions between active MOB and other microbes were assessed by a correlation network-based analysis with datasets covering diverse environments where closely interacting members of a consortium can potentially alter the methane oxidation activity. Although, methanotrophy is used as a model system, the fundamentals of our postulations may be applicable to other microbial guilds mediating other biogeochemical processes.

## Introduction

Natural microbial communities are characterized by complex networks of microbial populations forming intricate relationships of synergistic, antagonistic, and/or neutral nature. Accumulating evidence stresses the relevance of microbial interactions and their role in altering microbial mediated processes, referred here as community/ecosystem functioning (Murase and Frenzel, 2008; Comolli, 2014; Daebeler et al., 2014; Ho et al., 2014; Abrudan et al., 2015; Amin et al., 2015; Fiegna et al., 2015; Willett et al., 2015). Moreover, emergent properties may arise when microorganisms interact (e.g., interaction-induced production of metabolites; Watrous et al., 2012; Tyc et al., 2014; Abrudan et al., 2015), leading to altered community functions otherwise absent in the case of non-interacting individual cells. Therefore, biotic interactions can be important modulators in community functioning, steering the community composition and dynamics. As such, determining significant relatedness between responses of microbial communities to specific environmental cues, and/or linking the diversity (evenness and richness) and abundance to process rates, without taking underlying biotic interactions into account, may lead to misguided views on causal relationships as well as an incomplete understanding of ecosystem functioning.

Interdependent relationships between microorganisms due to nutritional reliance between community members are well known (e.g., symbiotic phototrophic consortia comprising green sulfur bacteria and members of *Betaproteobacteria*; Müller and Overmann, 2011). In contrast, exemplified by aerobic methane-oxidizing bacteria (MOB), we focused on microorganisms which are regarded as being able to function as individuals without relying on any interacting partners for growth (see review by Semrau et al., 2010 for MOB metabolism). However, it has been shown *in vitro* that the presence of other microorganisms may still significantly alter process rates (i.e., methane oxidation as the functional response variable; Iguchi et al., 2011; Ho et al., 2014; Jeong et al., 2014; Oshkin et al., 2014), prompting us to hypothesize that microbial interactions in complex communities modulate process rates, and may account for observed variability in biogeochemical processes. Here, we find evidence for the relevance of microbial interaction in modulating biogeochemistry by reviewing the literature for close associations of MOB with their biotic neighbors which may lead to altered methane oxidation rates. Support for possible consistent metabolic interactions in methane-fed communities spanning multiple habitats was inferred using co-occurrence network analysis of selected datasets where methane-derived carbon incorporation into MOB and non-MOB community members was performed by combining DNA-based stable isotope probing (SIP), and high throughput sequencing (Table 1). The coupling of ^13^C–CH_4_ labeling to the network analysis allows the assessment of associations of actively interacting microorganisms, sharing carbon derived from a single relevant biogeochemical process (Dumont et al., 2011). However, our co-occurrence network analysis comes with a caveat; we cannot assess the spatial and temporal dynamics of the interaction as the DNA-SIP studies were only performed at a particular point in space and time. Moreover, these interactions may be affected by edaphic properties of the soil/sediment from the different environments. Phylogenetic assignment and relative abundance of community members in these datasets were determined *de novo* using a standardized pipeline (see Supplementary Information), enabling the comparison and interpretation of networks built for different environments revealing (in) consistent associations of MOB with other microbes. We argue that incorporating mechanistic knowledge on biotic interactions in community functioning is a step forward in linking microbial diversity and abundance to ecosystem functioning, facilitating predictions of ecosystem functioning under disturbance.

**Table 1:** **Studies considered for the network analysis, including site information and incubation/experimental conditions**.

**Habitat**	**Location (sampling time)**	**Methodology**	**Incubation conditions**					**Placement of network analysis and OTU table**
			**Incubation period (d)**	**Temperature (°C)**	**Headspace methane (% _v/v_)**	**Treatment**	**References**	
Sediment from geothermal springs	Hot springs across Canada (2009–2012)	SIP coupled to 16 s rRNA gene sequencing	7	22–45 (*in situ* temperature)	5–10	Un-amended incubations.	Sharp et al., 2014	Figure 1, Table S1
Sediment from a freshwater lake^#^	Lake Qalluuraq, Alaska, USA (July, 2009)	SIP coupled to 16 s rRNA gene sequencing	212–248 144–212 55–74	4 10 21	10	Un-amended incubation of sediments (0–1 and 15–20 cm from surface).	He et al., 2012a	Figure S2, Table S2
			188	4	10	Un-amended incubation of sediment (0–1 cm from surface).	He et al., 2012b	
			38–212	4 10 21	10	Un-amended incubation of water column and sediment (0–25 cm from surface).	He et al., 2012c	
Grassland soil	Grændalur Valley, Iceland (August, 2012)	SIP coupled to 16 s rRNA gene sequencing	28	25	1	Un-amended and amended oxic incubations with 15 and 150 μg ${\text{NH}}_{4}^{+}$−N g dw^−1^.	Daebeler et al., 2014	Figure S3, Table S3
Rice paddy soil	Jiangsu Province, China (January, 2009)	SIP coupled to 16 s rRNA gene sequencing	19	28	0.9–1	Amended oxic incubations with CH_4_, CH_4_+urea, and CH_4_+urea+ CO_2_.	Zheng et al., 2014	Figure S4, Table S4
Surface water of oilsands tailing pond	Fort McMurray, Alberta, Canada (2010–2011 at 3 months intervals)	SIP coupled to 16 s rRNA gene sequencing	6–10	23	1	Oxic incubation with CO_2_ adjusted to 10 %_v/v_	Saidi-Mehrabad et al., 2013	Figure S5, Table S5

# *The network analysis was derived from three studies of the same environment (by the same main authors)*.

## MOB form close associations with their biotic environment

Aerobic methane-oxidizing bacteria co-exist with other (micro)organisms, and actively interact to form tight associations with their biotic environment. A mutually beneficial interaction occurs in ombrotrophic peatlands where MOB-*Sphagnum* moss interaction is thought to drive carbon sequestration by *Sphagnum*, while mitigating methane emission (Putkinen et al., 2012; Larmola et al., 2014; Vile et al., 2014). Diazotrophic MOB seemingly form a symbiotic relationship with *Sphagnum*; in return for molecular oxygen, the MOB provide the moss with additional carbon in the form of CO_2_ derived from the respired methane, as well as being a source for assimilable nitrogen by MOB nitrogen fixation (Raghoebarsing et al., 2005; Larmola et al., 2014; Vile et al., 2014; Kox et al., 2016). Given the close proximity of the MOB being localized in the hyaline cells of the *Sphagnum* and that nitrogen fixation is energetically costly, it has recently been proposed that a more mutually beneficial partnership yielding a higher return on investment (e.g., reducing equivalents needed in methane oxidation) for the MOB may occur (Ho and Bodelier, 2015). Therefore, the MOB are suggested to be closely associated to the *Sphagnum*, which forms the base of the food web in peat ecosystems in an inter-play inherent to the carbon and nitrogen cycles in peatlands. Similarly, in a stratified lake (Lago di Cadagno, Switzerland) where light penetrated to the anoxic zone, aerobic methane oxidation was fuelled by molecular oxygen produced *in situ* by photosynthetic algae (Milucka et al., 2015). Not only were gammaproteobacterial MOB found to form the active population assimilating methane in this environment, these microorganisms were also expressing the *nifH* gene, a subunit of the gene encoding for the nitrogenase enzyme, indicating their likely contribution to the carbon and nitrogen cycles (Halm et al., 2009; Milucka et al., 2015). In a partnership of microalgae and MOB, the microalgae were also found to fuel aerobic methane oxidation under oxygen-limiting conditions (Van der Ha et al., 2011). Indirect interaction with invertebrates can be seen in a termite mound where the termites engineer their immediate environment, shaping the MOB community composition and significantly stimulated methane oxidation (Ho et al., 2013a). Likewise, in the marine environment, MOB act as epibiont/endosymbiont of benthic invertebrates around hydrothermal vents, a hotspot for methane cycling. For instance, gammaproteobacterial MOB were found to be part of the active epibiotic community in the setae of *Shinkaia crosniere*, a deep sea dwelling crab found around hydrothermal vents (Watsuji et al., 2014). In a stable isotope labeling study, ^13^C derived from ^13^C–CH_4_ to H^13^${\text{CO}}_{3}^{-}$ (bicarbonate) could be retrieved from tissue of the *S. crosniere*, showing that carbon derived from MOB or other epibionts was assimilated into the crab, and provided evidence that epibionts may also nutritionally support their host, a role that was so far only evident in endosymbionts (Watsuji et al., 2010). Among the epibiotic community in the hydrothermal shrimp *Rimicaris exoculata*, gammaproteobacterial MOB were found to be localized in the gill chamber of the shrimp (Zbinden et al., 2008). Specific localization of methanotrophic epibionts was also demonstrated for a hydrothermal vent mussel *Bathymodiolus puteoserpentis* where higher *pmoA* gene expression was detected in areas where methane was transported into the mussel by water flow (Wendeberg et al., 2012); *pmoA* gene expression was higher in the frontal regions of the gill, and decreased toward the anterior. Although the exact role of MOB in many of these interactions require further mechanistic probing, it is clear that MOB form significant relationships with their biotic components in widespread environments.

Moreover, MOB may benefit from interaction with other prokaryotes. In a microbial community enriched from a forest soil, specific heterotrophs (*Rhizobium* sp.) are thought to provide MOB with essential nutrients (Iguchi et al., 2011). Co-culturing MOB along with some *Rhizobium* sp. increased the growth of *Methylovulum miyakonense*, an alphaproteobacterial MOB. Analyzing the filtered spent medium from the co-culture identified the growth-stimulating factor to be cobalamin, an essential trace nutrient the MOB are incapable of synthesizing intracellularly, and thus rely on an external source; the *Rhizobium* seemingly provides these MOB with cobalamin, stimulating growth (Iguchi et al., 2011). Similarly, other microorganisms may promote growth of MOB (e.g., *Cupriavidus taiwanensis*: Stock et al., 2013; *Sphingopyxis* sp: Jeong et al., 2014). In the MOB—*Sphingopyxis* sp co-culture, significant stimulation of methanotrophic activity and growth was attributable to higher gene transcription of the enzymes involved in methane catabolism (Jeong et al., 2014). Conversely, MOB are known to sustain entire isolated ecosystems and act as a primary producer in methane-driven environments (e.g., Movile cave, Romania; Hutchens et al., 2004: aquatic ecosystems; Agasild et al., 2014), as well as in enrichments with methane as the sole carbon and energy source (Beck et al., 2013; Oshkin et al., 2014). In these enrichments, however, specific accompanying microorganisms (e.g., methylotrophic species; Beck et al., 2013; Kerckhof et al., 2014; Oshkin et al., 2014; Yu et al., 2016) were observed to co-enrich along with the MOB even after successive transfers in independent studies, indicating a reciprocal selection of MOB and non-methanotrophic interacting partners. In a soil and lake sediment from the Arctic region, the relative abundance of methanotrophs and methylotrophs was directly correlated, suggesting a close association of these groups of microorganisms, likely as a consequence of a direct exchange of metabolites (Martineau et al., 2010; He et al., 2012a). The nature of the interaction remains enigmatic, but is worthy of speculation (see below). Besides, recently discovered versatility in the MOB metabolism shows that *Methylomicrobium alcaliphilum*, an obligate gammaproteobacterial MOB may directly exude carbon-based compounds (e.g., acetate, succinate) under oxygen limitation (Kalyuzhnaya et al., 2013), suggesting yet another means by which MOB can support heterotrophic microorganisms. Although these studies suggest that MOB interact with specific microorganisms, MOB interacting partners may not be necessarily exclusive (Ho et al., 2014). On the contrary, Ho et al. (2014) showed the relevance of having a diverse microbial community, regardless of the community members, to significantly stimulate methanotrophic activity. Collectively, these studies demonstrate a close-knit association of MOB and other biotic components across multi-trophic levels.

Not all interactions are cooperative/synergistic endeavors (Oliveira et al., 2014). Antagonistic biotic interactions can be represented by a predator-prey relationship between MOB and protists where gammaproteobacterial MOB were found to be preferentially grazed than alphaproteobacterial ones (Murase and Frenzel, 2008). Protist grazing caused a shift in the soil bacterial community composition, including the MOB (Murase et al., 2006; Murase and Frenzel, 2007), and is thought to affect methane oxidation as a consequence of an indirect effect through enhanced nitrogen mineralization following grazing (Murase and Frenzel, 2007). Similarly, a predatory relationship had been suggested for the interaction between the MOB *Methylocapsa acidiphila*, and the white rot fungus *Hypholoma fasciculare* (De Boer and van der Wal, 2008). The presence of *H. fasciculare* in beech wood coincided with the reduction of wood-inhabiting bacteria due to bactericidal effects induced by the fungus. Among the bacteria still detected at a relatively high proportion in the beech wood was *M. acidiphila*, an acidophilic MOB capable of N-fixation, which can utilize both methane and methanol as substrates (Dedysh et al., 2002). Hence, this may seem like a mutualistic interaction where the MOB provide nitrogen in return for methanol (a side-product of ligninolytic activity), but the authors also suggest a predatory interaction (induced bactericidal effect to gain assimilable nitrogen from lysed cells; De Boer and van der Wal, 2008). Further support for possible antagonistic interactions between MOB and fungi can be found in a correlative study showing spatial and/or niche separation between MOB and fungal abundances (Burke et al., 2012). Therefore, both synergistic and antagonistic interactions determine MOB distribution and prevalence in the environment, with possible consequences for environmental methane oxidation.

## Biotic interaction modulating MOB activity

**Figure 1:**
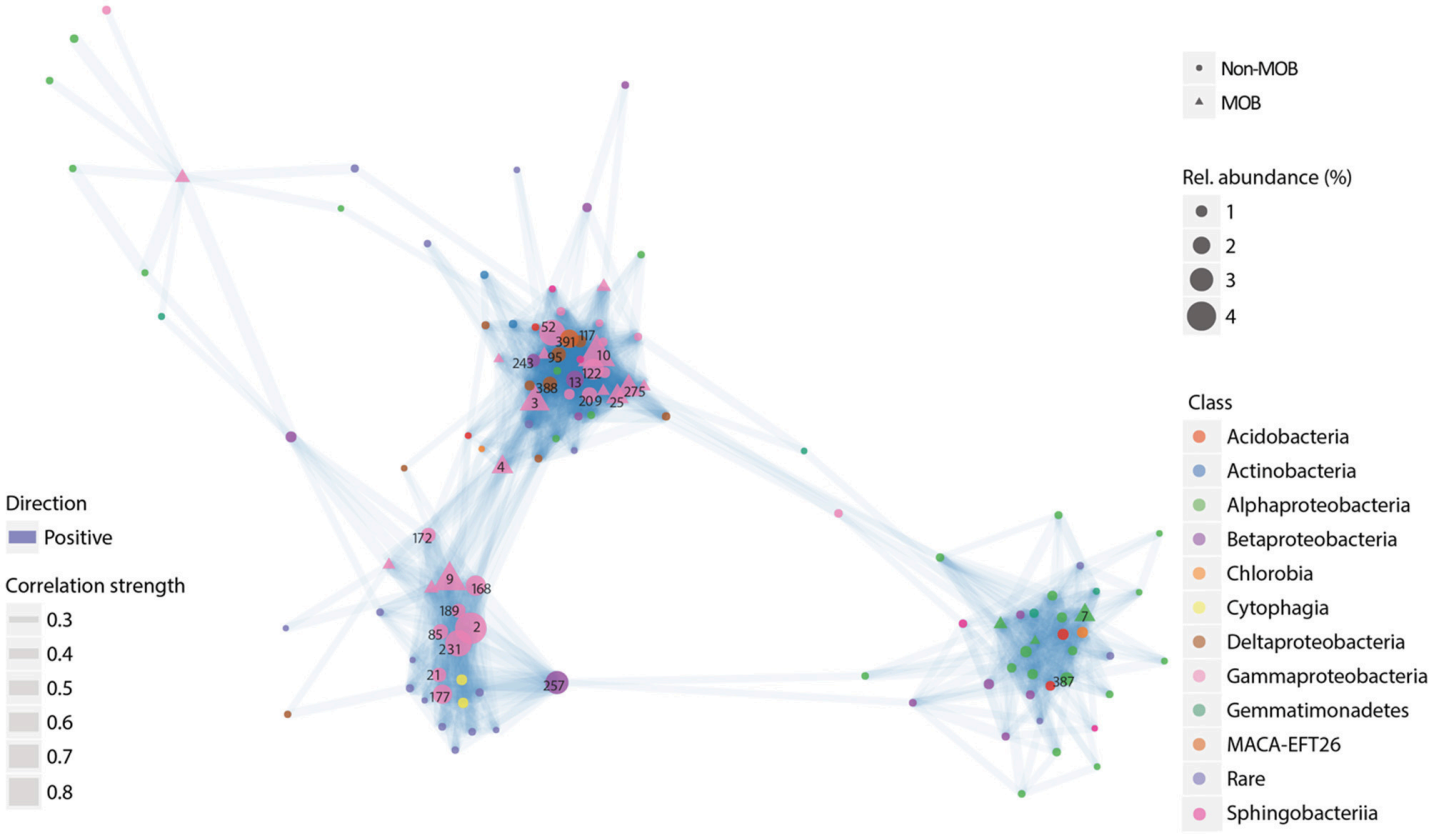
**Representative co-occurrence network of OTUs derived from 16 s rRNA gene sequences**. The network depicts OTUs classified as MOB together with other OTUs which significantly and positively correlated with them. The OTUs were derived from the “heavy” fraction (i.e., isotopically labeled DNA) of a SIP gradient from a ^13^C–CH_4_ labeling experiment of a microbial community in sediments from a geothermal spring (Sharp et al., 2014). Only OTUs with >10 total reads and which appeared in >20% of the samples were taken into account. Full taxonomic affiliation corresponding to the numbers are listed in the Supplementary Information (Table S1). The experimental conditions and site information are given in Table 1.

Metabolites are the currency of microbial interaction (Morris et al., 2013; Beliaev et al., 2014; Amin et al., 2015; Audrain et al., 2015; Schmidt et al., 2015; Zelezniak et al., 2015). In broad terms, metabolites encompass (by) products of microbial metabolism, including (non) volatile compounds which can be secreted into the environment, inducing interaction with synergistic, antagonistic, and neutral outcomes. Elucidating the link between microbial interaction in natural communities and biogeochemical processes is made challenging by the complexity of the potential metabolic networks between co-occurring microorganisms. Moreover, it is not trivial to pinpoint the source of a particular metabolite given the versatility in microbial metabolism which confers a high level of redundancy to a single process. Therefore, we focused on methane-driven environments to find evidence for putative, and possibly, consistent interactions, as represented in single resource driven communities. Accordingly, we surveyed the literature for ^13^C–CH_4_ labeling studies (Table 1), and mined datasets derived from these studies to perform network analyses on the microbial communities (derived from the 16S rRNA gene) incorporating the ^13^C (Figure 1, Figures S2–S5; see Supplementary Information for details on network construction). In contrast to previous work inferring interaction *via* coexistence of microbial communities in DNA-based studies (excluding SIP), the coupling of ^13^C–CH_4_ labeling to a network analysis provides a direct link through a shared substrate and cross-feeding between interacting microorganisms. Hence, while previous work provided direct evidence for interaction-induced modulation of methane oxidation in simplified ecosystems (i.e., synthetic communities; Iguchi et al., 2011; Stock et al., 2013; Ho et al., 2014; Jeong et al., 2014), our present approach combining DNA-SIP and a co-occurrence network analysis provides a first insight into the MOB interactome of naturally-occurring complex communities.

Based on the literature survey, we selected seven datasets (Table 1) covering widespread terrestrial methane-cycling environments (i.e., arctic lake, geothermal springs, oilsands tailings ponds, grassland, and rice paddy), and with adequate sequencing coverage to obtain sequences which could be affiliated to microorganisms at a high phylogenetic resolution (genus level). Co-occurrence networks were constructed using the OTU tables based on SparCC correlation coefficients (Friedman and Alm, 2012) and calculated in R (V3.2.2; R Core Team, 2014) using igraph (V1.0.1; Csardi and Nepusz, 2006). Briefly, the OTU tables were generated from raw sequence reads using Mothur (Schloss et al., 2009) and the UPARSE pipeline (Edgar, 2013), and classified against the SILVA NR 99 database (V119; Pruesse et al., 2007; see Supplementary Information). Besides, we only considered the ^13^C-labeled OTUs which represent >1% relative abundance assuming these to be the ones increased in abundance as a consequence of methane-derived carbon transfer.

The network analysis revealed a methane-derived food web in the study sites, and that, with the exception of the methylotrophs, the accompanying non-MOB community (taxonomic hierarchy: family and genus) was rather site-specific and not consistent; habitat-overarching set of genera was associated with the MOB. Interestingly, in some study sites (geothermal springs and arctic lakes; Figure 1, Figure S2), the network analysis revealed clustering of gammaproteobacterial and alphaproteobacterial MOB, and their associated non-MOB communities, suggesting that MOB species have distinct associated communities. This may point to differences in amount and composition of metabolites exchanged by MOB species. The clustering according to MOB subgroups is not obvious in the grassland soil (Figure S3), but a higher connectivity (degree of connectedness) was observed among microorganisms associated to the alphaproteobacterial MOB, indicating more complex routes of transfer of metabolites or a higher diversity of MOB “compatible” microorganisms in this habitat. Similarly, there were no obvious clusters in the rice paddy soil, but a higher connectivity was observed among the gammaproteobacterial MOB (Figure S4). The dominant non-MOB community (>1% relative abundance) associated to either the alphaproteobacterial or gammaproteobacterial MOB appears to be distinct within the study sites with the exception of *Xanthomonadaceae* in the grassland soil, albeit different genera were associated to the different MOB subgroups (e.g., Figures S1B,C). Between the study sites, families *Anaerolineaceae* and *Caulobacteraceae* (represented by different genera) were found to be associated to both MOB subgroups (e.g., Figures S1B,C). Hence, *in lieu* of lower taxonomic ranks (family/genus level), we focused on the communities which appear to be specific to the gammaproteobacterial and alphaproteobacterial MOB at the order level where some members seemingly converged after incubation (Figure S1). For instance, in the arctic lake sediment and grassland soil, OTUs affiliated to *Rhizobiales* were associated with *Methylosinus* (alphaproteobacterial MOB), whereas the non-MOB community associated with gammaproteobacterial MOB was more diverse with *Pseudomonadales* occurring in both sites. Although the accompanying non-MOB community differs, this trend was consistent in the sediment from the geothermal spring where *Rhodospirillales* was the only dominant order (>1% relative abundance) associated to *Methylosinus* (Figure 1, Table S1). *Rhodospirillales* was also consistently found to be associated with the alphaproteobacterial MOB in other sites (Figure S1). Given that only gammaproteobacterial MOB were predominantly active in the oilsands tailing ponds, a clustering based on MOB subgroups was not observed (Figure S5). Admittedly, considering higher taxonomic ranks will render considerable overlaps in the community composition. Yet, out of 27 dominant OTUs (order level), only *Xanthomonadales* was found in all sites with the exception of the arctic lake sediment, indicating that the occurrence of the methane-fueled community was site-and MOB-species specific. The site-to-site variation of the methane-driven community is not unexpected considering the different soil/sediment edaphic characteristics and environmental conditions of the study sites spanning across three continents (Knief, 2015). However, we cannot completely exclude methodological artifacts inherent to PCR-based studies (arising from high throughput sequencing). Further studies are needed to determine the spatial and temporal dynamics of the interacting partners. Nonetheless, the clustering and association of distinct accompanying microorganisms to the gammaproteobacterial and alphaproteobacterial MOB, more evident in some sites (sediments from the arctic lake and geothermal springs) than others, suggest a selection of interacting partners, possibly through different amounts and/or types of metabolites excreted.

Additionally, despite the different incubation conditions and the length of incubation (Table 1), the network analysis revealed the co-occurrence of methanol-oxidizers (e.g., *Methylotenera, Methylobacterium, Methylobacillus, Methylohalomonas*) and MOB in all sites, which is in accordance with a previous study where a high relative abundance (up to 40–50% of total community; Oshkin et al., 2014) of methylotrophs (e.g., *Methylotenera, Methylophilus*) was found in a methane-enriched community. It is often hypothesized that the co-detection of methylotrophs and methanotrophs in SIP studies are caused by cross-feeding; the methylotrophs feed on the methanol derived from methane oxidation. While cross-feeding is likely a cause for the co-occurrence of methylotrophs and MOB, the detection of other active microorganisms associated to the different MOB subgroups within each site may not be a stochastic event, but supports the notion of a selection of specific accompanying community members (Oshkin et al., 2014), particularly after several transferring steps (Yu et al., 2016). However, the network analysis was derived from ^13^C–CH_4_ labeling studies representing a snapshot of the active community. Hence, while our meta-analysis lacks in temporal and spatial scales, and is limited by the availability of physico-chemical data, it provides a first insight into the active MOB interactome. We showed that combining DNA-SIP to a co-occurrence network analysis is a powerful tool to relate interaction of active microorganisms. This approach, when applied to well-designed experimentation in future studies will divulge the robustness of an interacting community as well as the mechanisms of interaction by tracking the labeled metabolites.

## Mode of MOB interaction

Co-occurrence networks in microbial ecology visualize the positive and/or negative correlations between all members of several microbial communities (typically OTUs), and help predicting ecological interactions (Faust and Raes, 2012). The network analysis, however, does not reveal the mode of the interaction. In ^13^C–CH_4_ labeling studies, all non-MOB are positively correlated with the MOB, benefiting from their association with the MOB (*via* cross-feeding), although an antagonistic interaction (nature of the interaction) may also occur (e.g., predation). Biotic interactions can exert a direct and/or indirect effect, modulating process rates (Murase and Frenzel, 2007; Daebeler et al., 2014; Ho et al., 2014), and possibly, structuring the microbial community composition (Murase et al., 2006; Murase and Frenzel, 2007; Yu et al., 2016). In direct interaction, MOB/satellite communities release metabolites which exert a direct response, either mutually benefiting or adversely affecting the interacting partner. Despite their proven ability to synthesize and exude (secondary) metabolites (e.g., acetate, succinate, lactate; Kalyuzhnaya et al., 2013; ectoine: Reshetnikov et al., 2006; Khmelenina et al., 2015; methanobactin: Kim et al., 2004), the role of these compounds in MOB interaction remains largely unexplored. In particular, volatile secondary metabolites, being able to exert an effect even across physical barriers, have yet unknown ways of eliciting a response in community functioning (Schmidt et al., 2015). Indeed, it was only recently that studies began to show the importance of some secondary metabolites expressed and detected only when microorganisms were co-cultured (Watrous et al., 2012; Tyc et al., 2014), suggesting an overlooked interaction-induced mechanism to produce/release compounds.

Conversely, these metabolites may accumulate to prohibitive levels for both the MOB and other microorganisms, such as in the case of hydroxylamine and methanol, intermediary compounds of ammonium and methane oxidation, respectively, which may inhibit methanotrophic activity (Bodelier and Laanbroek, 2004; Poret-Peterson et al., 2008; Bodelier, 2011). “One man's meat is another man's poison” (*sic*); because functional traits among MOB vary (Ho et al., 2013b; Hoefman et al., 2014), some MOB and accompanying members of the consortium may consume the inhibitory compounds, thereby relieving toxicity, and facilitate growth and activity of other microorganisms in an indirect interaction. Indeed, a cooperative endeavor to relief inhibition between MOB and methylotrophs, as well as heterotrophs has been inferred in enrichment and co-culture studies (i.e., Beck et al., 2013; Stock et al., 2013; Oshkin et al., 2014), as in our network analyses where methylotrophs were consistently shown to be dominant members of the accompanying community in all study sites. Therefore, two modes (direct and/or indirect) of interaction underlie community patterns and functioning.

**Figure 2:**
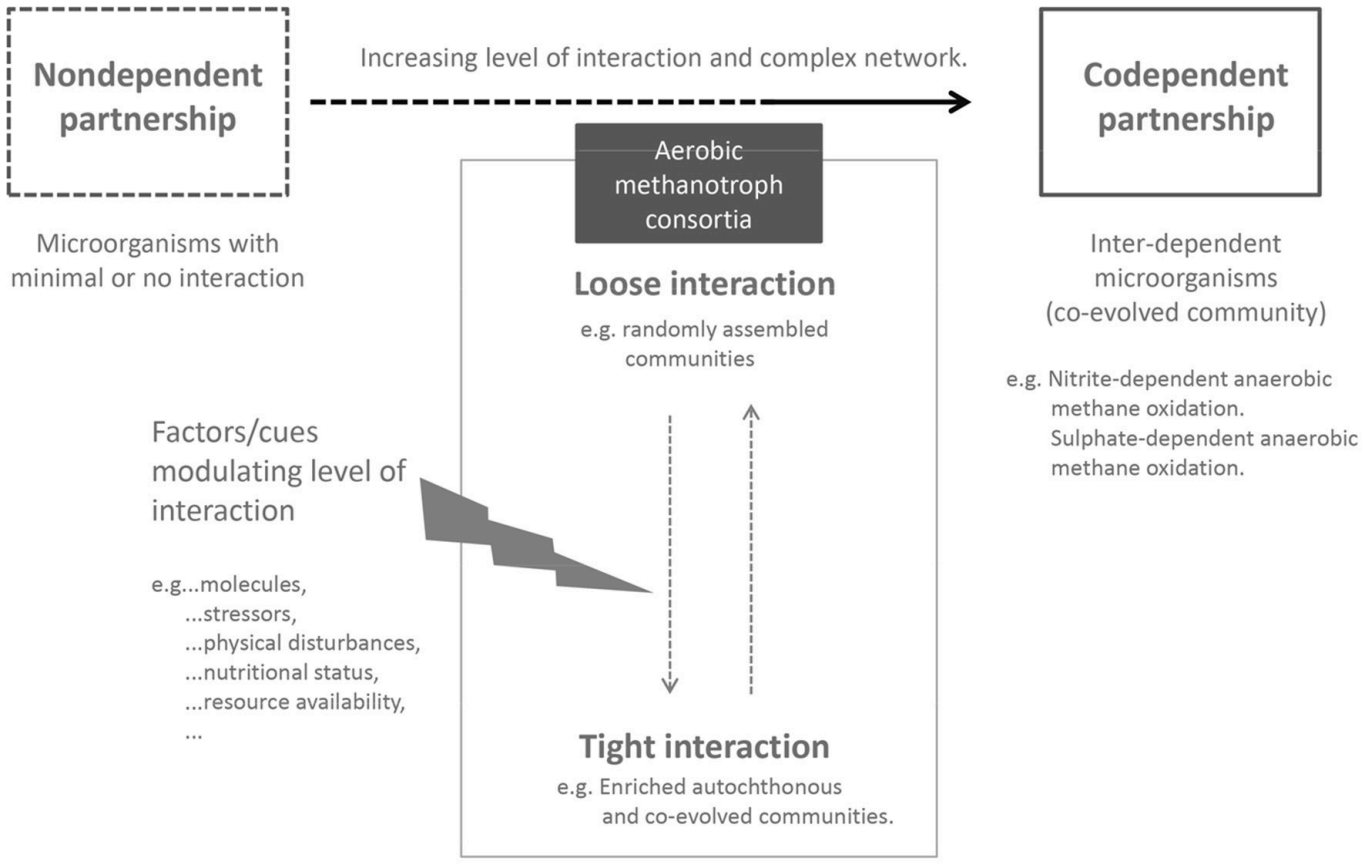
**Biotic interaction as modulator of methane oxidation**. Obligate aerobic MOB forms a close-knit community with its biotic component, benefiting from interaction with other microorganisms in the consortium. Yet, aerobic MOB are not dependent on the interacting microorganisms as depicted by a co-dependent partnership. Within the MOB consortia, the level of interaction may oscillate depending on environmental conditions and factors/cues affecting the community network.

Although, obligate MOB may not be solely dependent on other microorganisms, current understanding clearly shows their reliance on other interacting partners to facilitate survival and growth. To this end, our network analyses provide support for the potential selection of interacting partners specific to the different MOB subgroups. We postulate that MOB in interaction with their satellite microorganisms represent a close-knit association, but are not exclusive. Close cooperation (e.g., as a result of metabolic inter-dependencies) between microorganisms drives their co-occurrence (Fiegna et al., 2015; Zelezniak et al., 2015), which may evolve to become a co-dependent relationship (Figure 2; Morris et al., 2012). Exemplifying a potentially co-dependent interaction, the nitrite-dependent anaerobic methane oxidizer, *Candidatus* Methylomirabilis oxyfera has so far resisted purification but could be highly enriched (Ettwig et al., 2010). The gene cluster encoding the enzymes catalyzing the reduction of nitric oxide to molecular nitrogen and oxygen, a key pathway in the proposed scheme to self-oxygenate in *Ca*. M. oxyfera was undetected in the microorganism (Ettwig et al., 2010). Consequently, the authors suggest that the missing catalytic activity may be complemented by the action of other interacting partners in the enrichment. Such co-dependent interaction is true for another specialized process i.e., sulfate-dependent anaerobic methane oxidation (see reviews by Valentine and Reeburgh, 2000; Stams and Plugge, 2009). It stands to reason that a division of labor by splitting complex metabolic pathways or exchanging intermediate products (e.g., electron transfer; McGlynn et al., 2015) between multiple participants is a practical solution to overcome a lack of metabolic capacity and energetically demanding processes. An exception to the rule is cheater–microorganisms that exploit the cooperative interaction by imposing a cost on the cooperating partners, while benefiting themselves. The role of cheaters in the social behavior of microorganisms is recognized (Crespi, 2001), but is not yet firmly established in interacting MOB communities. On the other hand, the methane oxidation rate was significantly stimulated in co-cultures containing a high diversity of interacting partners although these accompanying microorganisms were randomly selected, and had not co-evolved with the methanotroph (Ho et al., 2014). Similarly, the combinations of methanotrophs and heterotrophs in a study showing a higher growth response in some co-cultures were randomly selected from a culture collection (Belgian Coordinated Collections of Microorganisms/Laboratory of Microbiology–Gent University, Gent, Belgium; Stock et al., 2013). Hence, synergistic microbial interactions may not necessarily be exclusive and restricted to co-evolved communities, which questions the predominant modes of interaction in different (co-evolved) communities.

## Concluding remarks

With emerging evidence, biotic interactions are gaining more recognition as important modulators of biogeochemical processes. However, similar to other attributes of microbial communities (e.g., diversity, traits), this “parameter” is not as well integrated in biogeochemical models designed to predict ecosystem functioning as well as processes (van de Leemput et al., 2011; Bouskill et al., 2012). Incorporation of explicit microbial traits into biogeochemical models, for example decomposition or greenhouse gas emission models, is starting to develop increasing predictive power (Treseder et al., 2012; Wieder et al., 2013, 2015; Wang et al., 2015) compared to traditional models. However, parameterizing of these models will require detailed knowledge on the breadth of trait responses and trade-offs in various microbial groups and processes which will very likely be strongly dependent on microbial interactions. The lack of this knowledge on traits and the role of biotic interactions in combination with the absence of a unifying framework to assess and determine when biotic interaction becomes relevant are the most important inadequacies, hindering integration of “biotic interaction” as a parameterized input in existing biogeochemical models. The association of ecosystem functioning with community diversity, traits, and abundances has been assessed *in vitro* based on experimental manipulation studies in the laboratory (Bell et al., 2005; Wertz et al., 2006; Wittebolle et al., 2009; Ho et al., 2011). Admittedly, microbial interactions underlie an array of relationships, shaping community composition, and although microbial interactions have been shown to be relevant controls of ecosystem functioning (Stock et al., 2013; Daebeler et al., 2014; Ho et al., 2014; Jeong et al., 2014), it remains a challenge to disentangle community interaction from other relationships (e.g., biodiversity-ecosystem functioning). Hence, microbial interaction is an integral component, often confounded, but seldom explicitly tested in complex communities particularly in the context of biogeochemical cycles. This reverberates previous calls for a more integrated approach, including microbial interaction when elucidating the response of community composition to environmental cues (Comolli, 2014; Lupatini et al., 2014). In complex environments, interaction may well be a key neglected determinant, if not as important as diversity and community abundance, driving ecosystem functioning. Hence, there is a need to move beyond our current understanding of relating biodiversity (richness and evenness) and abundance to ecosystem functioning in environmental studies; “biotic interaction” as a modulator of ecological processes warrants further attention.

Disentangling biotic interaction from other environmental parameters altering process rates is challenging. Nevertheless, this challenge may be partly circumvented by experimental setups capitalizing on artificially assembled communities (De Roy et al., 2013, 2014; Stenuit and Agathos, 2015). A synthetic community provides a well-defined biotic environment, allowing the assembly of communities comprising well-characterized microorganisms with available genomes, to reduce complexity in interaction. Therefore, synthetic communities facilitate understanding of the underlying mechanism of the interaction (e.g., bacteria–fungal interaction: Schneider et al., 2010; microbe–microbe interaction: Beliaev et al., 2014). However, because of the reduced complexity, it is not entirely surprising that community functioning in synthetic communities may not reflect on the behavior of naturally-occurring communities in the environment (Yu et al., 2016). Although, general compositional dynamics of the methanotrophs in synthetic communities have been shown to resemble dynamics of natural communities, the similarities were not observed at the species level (Yu et al., 2016). Nevertheless, knowledge gained from synthetic community studies may help predict community response. For instance, as shown by Stock et al. (2013), microbe-microbe interaction models can be “trained” (cross-validation techniques in supervised learning of predictive models; Hastie et al., 2009) to predict co-culture response. The input of the predictive model was derived from the growth response of a subset of MOB and heterotroph combinations, which was subsequently used for predicting all possible combinations of MOB and heterotrophs. *In lieu* of determining the growth response of all combinations of the co-cultures, the values were inferred using the predictive model (Stock et al., 2013).

Accordingly, microbial interaction may become important under certain conditions (Figure 2). For instance, a more complex microbial network may arise as a response to limiting substrate availability, forcing metabolic exchange and increase co-occurrence (Zelezniak et al., 2015). Moreover, there is a myriad of secondary (volatile) compounds secreted by microorganisms to the environment. These compounds may act as signaling molecules and have yet unknown ways of modulating process rates (Schulz-Bohm et al., 2015). For example, some secondary compounds (resuscitating-promoting factors; Lennon and Jones, 2011), as well as a shift in temperature (Ho and Frenzel, 2012; Ho et al., 2016) may awaken the dormant population, effectively contributing to the active members of a community. Only when we understand the mechanisms of interaction, can we predict the response of community functioning which calls for a strong focus on mechanistic studies using representative microbes, catalyzing a relevant biogeochemical process.

## Author contributions

AH, PB conceived the review and analysis. RA performed the analyses with input from AD, AH, and PB. AH wrote the initial manuscript. RA, AV, AD, ZJ, SK, FK, NB, and PB critically revised and approved the manuscript. All authors are accountable for all aspects of the work.

### Conflict of interest statement

The authors declare that the research was conducted in the absence of any commercial or financial relationships that could be construed as a potential conflict of interest.

## Supplementary material

Figure S1 **Venn diagrams showing co-occurring non-MOB community (order level) in all environments (A), as well as the accompanying non-MOB community associated to the alphaproteobacterial (B), and gammaproteobacterial (C) MOB where relevant**. MOB and unclassified microorganisms are not included in the Venn diagrams. Blue, green, orange, gray, and turquoise denote samples collected from a geothermal spring sediment, grassland soil, arctic lake sediment, oilsands tailing ponds sediment, and rice paddy soil, respectively.

Figure S2 **Network analysis of 16s rRNA gene sequences derived from the “heavy” fraction of a ^**13**^C-CH_**4**_ labeled community in sediments from an arctic lake (He et al., 2012a,b,c)**. The correlating OTUs with >1% relative abundance are given in the figure, and the corresponding taxonomic affiliation are listed in the Supplementary Information (Table S2).

Figure S3 **Network analysis of 16s rRNA gene sequences derived from the “heavy” fraction of a ^**13**^C-CH_**4**_ labeled community in a grassland soil (Daebeler et al., 2014)**. The correlating OTUs with >1% relative abundance are given in the figure, and the corresponding taxonomic affiliation are listed in the Supplementary Information (Table S3).

Figure S4 **Network analysis of 16s rRNA gene sequences derived from the “heavy” fraction of a ^**13**^C-CH_**4**_ labeled community in a rice paddy soil (Zheng et al., 2014)**. The correlating OTUs with >1% relative abundance are given in the figure, and the corresponding taxonomic affiliation are listed in the Supplementary Information (Table S4).

Figure S5 **Network analysis of 16s rRNA gene sequences derived from the “heavy” fraction of a ^**13**^C-CH_**4**_ labeled community in sediments from oilsands tailings ponds (Saidi-Mehrabad et al., 2013)**. The correlating OTUs with >1% relative abundance are given in the figure, and the corresponding taxonomic affiliation are listed in the Supplementary Information (Table S5).

Table S1 **Co-occurring OTUs with >1% relative abundance derived from the “heavy” fraction of a ^**13**^C-CH_**4**_ labeled community in sediments from geothermal springs**. Classification of OTUs is as given in Sharp et al. (2014). Bold and gray scripts denote MOB and methylotroph, respectively.

Table S2 **Co-occurring OTUs with >1% relative abundance derived from the “heavy” fraction of a ^**13**^C-CH_**4**_ labeled community in sediments from an arctic lake**. Classification of OTUs is as given in He et al. (2012a,b,c). Bold and gray scripts denote MOB and methylotroph, respectively.

Table S3 **Co-occurring OTUs with >1% relative abundance derived from the “heavy” fraction of a ^**13**^C-CH_**4**_ labeled community in a grassland soil**. Classification of OTUs is as given in Daebeler et al. (2014). Bold and gray scripts denote MOB and methylotroph, respectively.

Table S4 **Co-occurring OTUs with >1% relative abundance derived from the “heavy” fraction of a ^**13**^C-CH_**4**_ labeled community in a rice paddy soil**. Classification of OTUs is as given in Zheng et al. (2014). Bold and gray scripts denote MOB and methylotroph, respectively

Table S5 **Co-occurring OTUs with >1% relative abundance derived from the “heavy” fraction of a ^**13**^C-CH_**4**_ labeled community in oilsands tailings ponds**. Classification of OTUs is as given in Saidi-Mehrabad et al. (2013). Bold and gray scripts denote MOB and methanol-oxidizer, respectively.
