# Supplementary material for: Biotic Interactions in Microbial Communities as Modulators of Biogeochemical Processes: Methanotrophy as a Model System
Source: Front Microbiol. 2016 Aug 23;7:1285. doi: 10.3389/fmicb.2016.01285 (PMC4993757; doi:10.3389/fmicb.2016.01285)
Supplement: Figure S1 — Venn diagrams showing co-occurring non-MOB community (order level) in all environments (A), as well as the accompanying non-MOB community associated to the alphaproteobacterial (B), and gammaproteobacterial (C) MOB where relevant. MOB and unclassified microorganisms are not included in the Venn diagrams. Blue, green, orange, gray, and turquoise denote samples collected from a geothermal spring sediment, grassland soil, arctic lake sediment, oilsands tailing ponds sediment, and rice paddy soil, respectively. [file Image1.PDF]

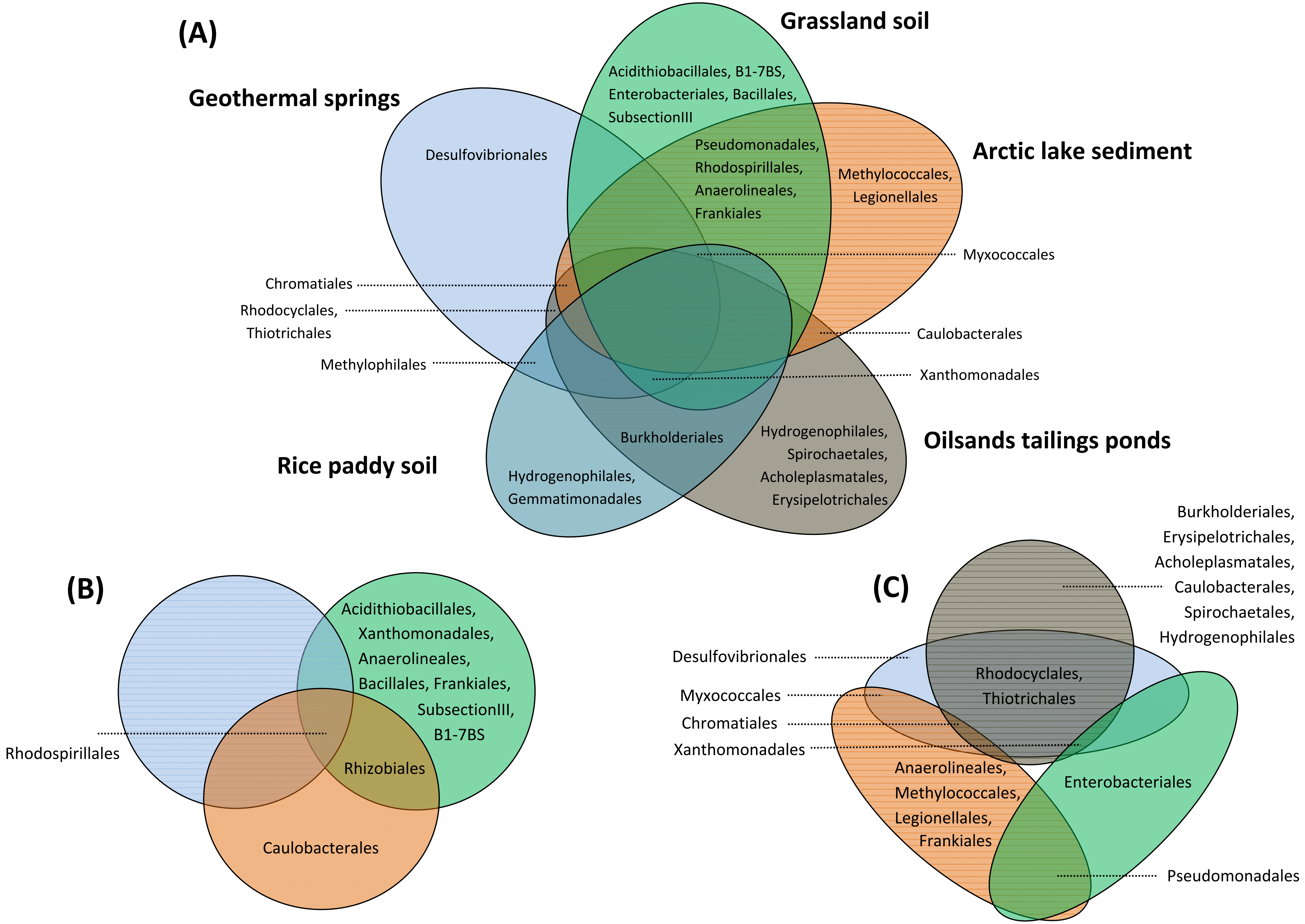
(A)
Grassland soil
Geothermal springs
Arctic lake sediment
Oilsands tailings ponds
Rice paddy soil
Acidithiobacillales, B1-7BS,
Enterobacteriales, Bacillales,
SubsectionIII
Desulfovibrionales
Pseudomonadales,
Rhodospirillales,
Anaerolineales,
Frankiales
Methylococcales,
Legionellales
Myxococcales
Chromatiales
Rhodocyclales,
Thiotrichales
Caulobacterales
Methylophilales
Xanthomonadales
Burkholderiales
Hydrogenophilales,
Spirochaetales,
Acholeplasmatales,
Erysipelotrichales
Hydrogenophilales,
Gemmatimonadales
(B)
Acidithiobacillales,
Xanthomonadales,
Anaerolineales,
Bacillales, Frankiales,
SubsectionIII,
B1-7BS
Rhodospirillales
Rhizobiales
Caulobacterales
(C)
Burkholderiales,
Erysipelotrichales,
Acholeplasmatales,
Caulobacterales,
Spirochaetales,
Hydrogenophilales
Desulfovibrionales
Rhodocyclales,
Thiotrichales
Myxococcales
Chromatiales
Xanthomonadales
Anaerolineales,
Methylococcales,
Legionellales,
Frankiales
Enterobacteriales
Pseudomonadales
