# Supplementary figures and images for: Biotic Interactions in Microbial Communities as Modulators of Biogeochemical Processes: Methanotrophy as a Model System
Source: Front Microbiol. 2016 Aug 23;7:1285. doi: 10.3389/fmicb.2016.01285 (PMC4993757; doi:10.3389/fmicb.2016.01285)

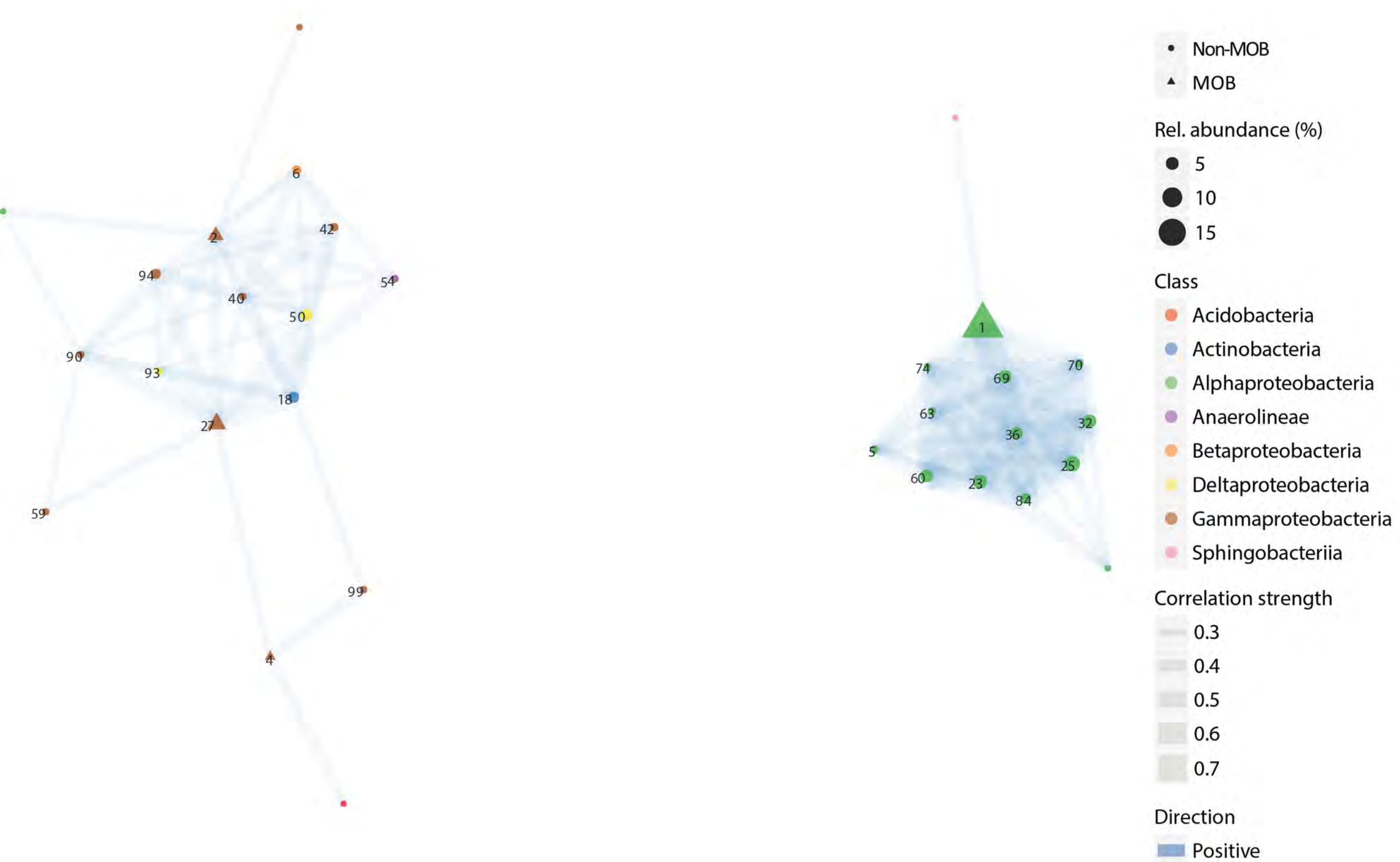

Supplement: Figure S2 — Network analysis of 16s rRNA gene sequences derived from the “heavy” fraction of a 13C-CH4 labeled community in sediments from an arctic lake (He et al., 2012a,b,c). The correlating OTUs with >1% relative abundance are given in the figure, and the corresponding taxonomic affiliation are listed in the Supplementary Information (Table S2). [file Image2.PDF]

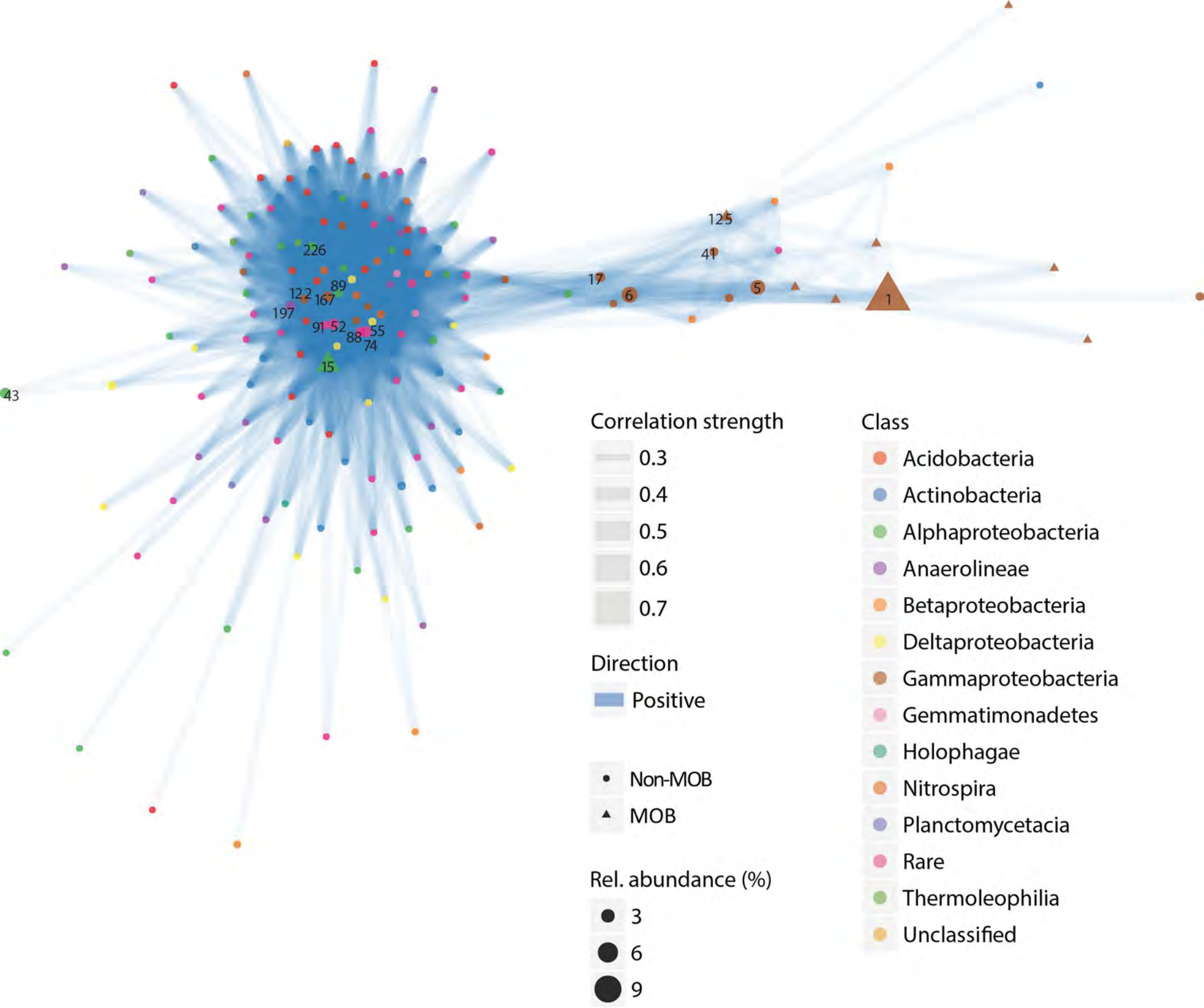

Supplement: Figure S3 — Network analysis of 16s rRNA gene sequences derived from the “heavy” fraction of a 13C-CH4 labeled community in a grassland soil (Daebeler et al., 2014). The correlating OTUs with >1% relative abundance are given in the figure, and the corresponding taxonomic affiliation are listed in the Supplementary Information (Table S3). [file Image3.PDF]

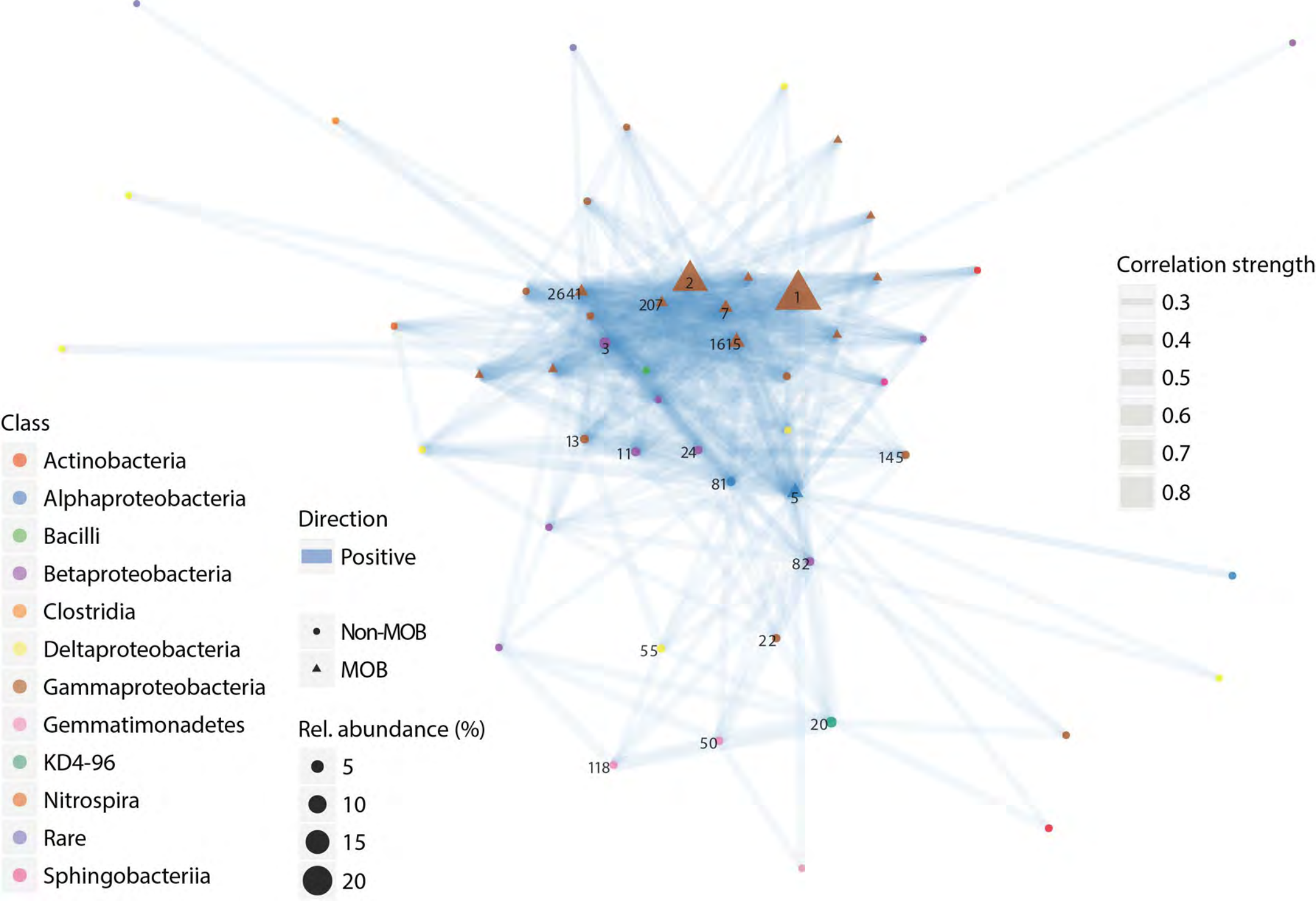

Supplement: Figure S4 — Network analysis of 16s rRNA gene sequences derived from the “heavy” fraction of a 13C-CH4 labeled community in a rice paddy soil (Zheng et al., 2014). The correlating OTUs with >1% relative abundance are given in the figure, and the corresponding taxonomic affiliation are listed in the Supplementary Information (Table S4). [file Image4.PDF]

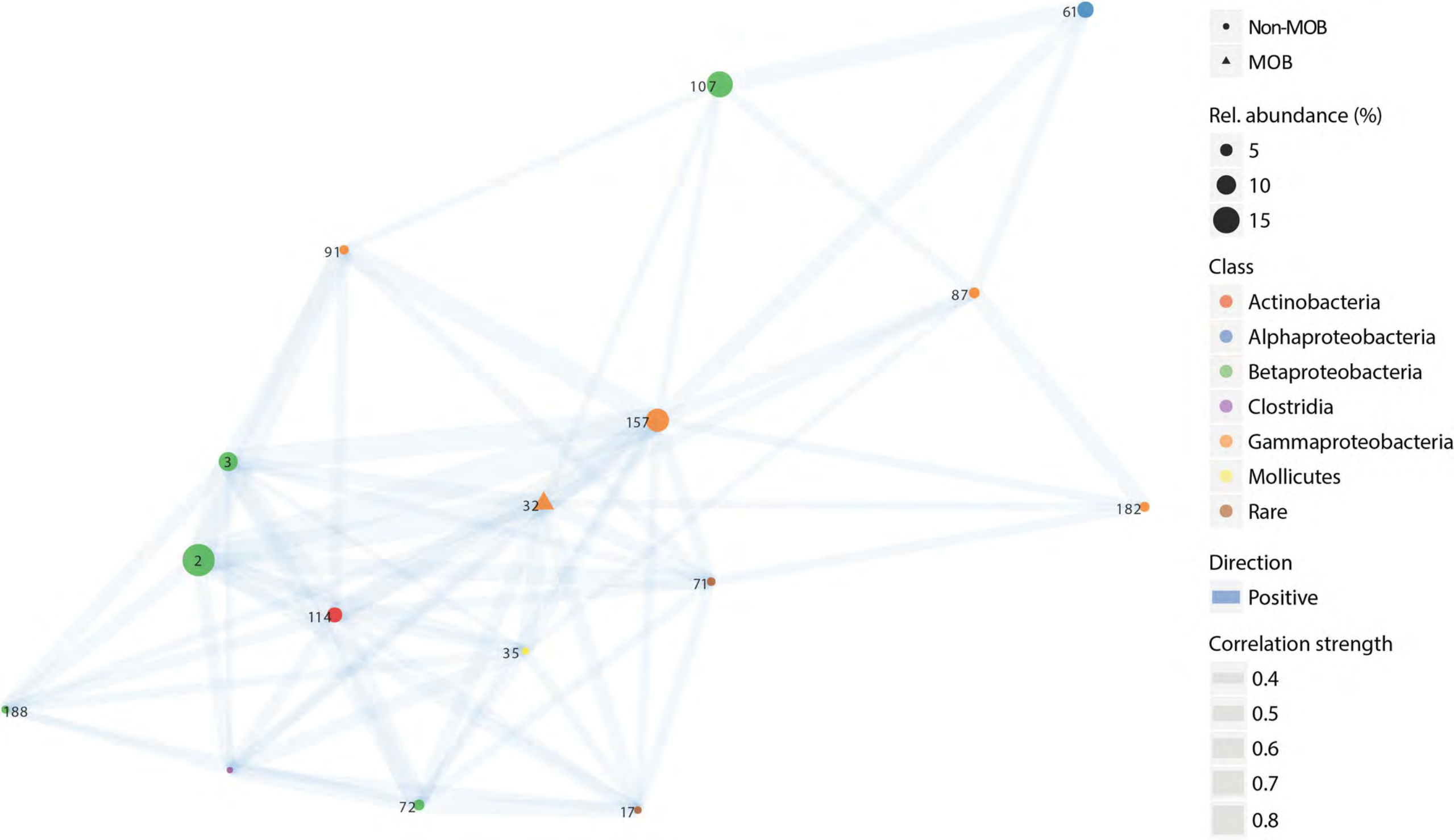

Supplement: Figure S5 — Network analysis of 16s rRNA gene sequences derived from the “heavy” fraction of a 13C-CH4 labeled community in sediments from oilsands tailings ponds (Saidi-Mehrabad et al., 2013). The correlating OTUs with >1% relative abundance are given in the figure, and the corresponding taxonomic affiliation are listed in the Supplementary Information (Table S5). [file Image5.PDF]
