# Supplementary material for: Biotic Interactions in Microbial Communities as Modulators of Biogeochemical Processes: Methanotrophy as a Model System
Source: Front Microbiol. 2016 Aug 23;7:1285. doi: 10.3389/fmicb.2016.01285 (PMC4993757; doi:10.3389/fmicb.2016.01285)
Supplement: Table S1 — Co-occurring OTUs with >1% relative abundance derived from the “heavy” fraction of a 13C-CH4 labeled community in sediments from geothermal springs. Classification of OTUs is as given in Sharp et al. (2014). Bold and gray scripts denote MOB and methylotroph, respectively. [file Table1.DOCX]

**Table S1**: Co-occurring OTUs with >1 % relative abundance derived from the ‘heavy’ fraction of a ^13^C-CH_4_ labeled community in sediments from geothermal springs. Classification of OTUs is as given in Sharp et al. (2014). Bold and grey scripts denote MOB and methylotroph, respectively.

| OTU  (~relative abundance) | Phyla | Class | Order | Family | Genus |
| --- | --- | --- | --- | --- | --- |
| 2 (4%) | Proteobacteria | Gammaproteobacteria | Thiotrichales | Thiotrichaceae | Beggiatoa |
| **3 (3%)** | **Proteobacteria** | **Gammaproteobacteria** | **Methylococcales** | **Methylococcaceae** | **Methylomonas** |
| **4 (2%)** | **Proteobacteria** | **Gammaproteobacteria** | **Methylococcales** | **Methylococcaceae** | **Methylocaldum** |
| **7 (3%)** | **Proteobacteria** | **Alphaproteobacteria** | **Rhizobiales** | **Methylocystaceae** | **Methylosinus** |
| **9 (4%)** | **Proteobacteria** | **Gammaproteobacteria** | **Methylococcales** | **Methylococcaceae** | **Methylocaldum** |
| **10 (>4%)** | **Proteobacteria** | **Gammaproteobacteria** | **Methylococcales** | **Methylococcaceae** | **Methylomonas** |
| 13 (2%) | Proteobacteria | Betaproteobacteria | Methylophilales | Methylophilaceae | Methylotenera |
| 21 (2%) | Proteobacteria | Gammaproteobacteria | 1013-28-cG33 | Unclassified | Unclassified |
| **25 (2%)** | **Proteobacteria** | **Gammaproteobacteria** | **Methylococcales** | **Methylococcaceae** | **Methylobacter** |
| 52 (4%) | Proteobacteria | Gammaproteobacteria | Chromatiales | Ectothiorhodospiraceae | Acidiferrobacter |
| 85 (2%) | Proteobacteria | Gammaproteobacteria | Xanthomonadales | Xanthomonadales Incertae Sedis | Steroidobacter |
| 95 (2%) | Proteobacteria | Deltaproteobacteria | Desulfovibrionales | Desulfohalobiaceae | Desulfothermus |
| 117 (1%) | Proteobacteria | Deltaproteobacteria | Myxococcales | Mle1-27 | Unclassified |
| 122 (3%) | Proteobacteria | Gammaproteobacteria | Xanthomonadales | Xanthomonadaceae | Arenimonas |
| 168 (3%) | Proteobacteria | Gammaproteobacteria | Thiotrichales | Thiotrichaceae | Methylohalomonas |
| 172 (2%) | Proteobacteria | Gammaproteobacteria | Chromatiales | Ectothiorhodospiraceae | Alkalispirillum |
| 177 (2%) | Proteobacteria | Gammaproteobacteria | Chromatiales | Ectothiorhodospiraceae | Acidiferrobacter |
| 189 (1%) | Proteobacteria | Gammaproteobacteria | Xanthomonadales | Xanthomonadaceae | Panacagrimonas |
| 209 (2%) | Proteobacteria | Gammaproteobacteria | Chromatiales | Ectothiorhodospiraceae | Acidiferrobacter |
| 231 (4%) | Proteobacteria | Gammaproteobacteria | Thiotrichales | Thiotrichaceae | Beggiatoa |
| 243 (1%) | Proteobacteria | Betaproteobacteria | B1_7BS | Unclassified | Unclassified |
| 257 (3%) | Proteobacteria | Betaproteobacteria | Rhodocyclales | Rhodocyclaceae | Thauera |
| **275 (2%)** | **Proteobacteria** | **Gammaproteobacteria** | **Methylococcales** | **Methylococcaceae** | **Methylomonas** |
| 387 (2%) | Proteobacteria | Alphaproteobacteria | Rhodospirillales | Rhodospirillales Incertae Sedis | Ca. Alysiosphaera |
| 388 (2%) | Proteobacteria | Deltaproteobacteria | Myxococcales | Haliangiaceae | Haliangium |
| 391 (2%) | Proteobacteria | MACA-EFT26 | Unclassified | Unclassified | Unclassified |
