# Supplementary material for: Biotic Interactions in Microbial Communities as Modulators of Biogeochemical Processes: Methanotrophy as a Model System
Source: Front Microbiol. 2016 Aug 23;7:1285. doi: 10.3389/fmicb.2016.01285 (PMC4993757; doi:10.3389/fmicb.2016.01285)
Supplement: Table S2 — Co-occurring OTUs with >1% relative abundance derived from the “heavy” fraction of a 13C-CH4 labeled community in sediments from an arctic lake. Classification of OTUs is as given in He et al. (2012a,b,c). Bold and gray scripts denote MOB and methylotroph, respectively. [file Table2.DOCX]

**Table S2**: Co-occurring OTUs with >1 % relative abundance derived from the ‘heavy’ fraction of a ^13^C-CH_4_ labeled community in sediments from an arctic lake. Classification of OTUs is as given in He et al. (2012a,b,c). Bold and grey scripts denote MOB and methylotroph, respectively.

| OTU  (~relative abundance) | Phyla | Class | Order | Family | Genus |
| --- | --- | --- | --- | --- | --- |
| **1 (> 15%)** | **Proteobacteria** | **Alphaproteobacteria** | **Rhizobiales** | **Methylocystaceae** | **Methylosinus** |
| **2 (10%)** | **Proteobacteria** | **Gammaproteobacteria** | **Methylococcales** | **Methylococcaceae** | **Methylobacter** |
| **4 (5%)** | **Proteobacteria** | **Gammaproteobacteria** | **Methylococcales** | **Methylococcaceae** | **Methylosoma** |
| 5 (<5%) | Proteobacteria | Alphaproteobacteria | Rhizobiales | Hyphomicrobiaceae | Hyphomicrobium |
| 6 (<5%) | Proteobacteria | Betaproteobacteria | DR-16 | Unclassified | Unclassified |
| 18 (5%) | Actinobacteria | Actinobacteria | Frankiales | Sporichthyaceae | Hgcl clade |
| 23 (5%) | Proteobacteria | Alphaproteobacteria | Rhizobiales | Rhizobiales Incertae Sedis | Rhizomicrobium |
| 25 (>5%) | Proteobacteria | Alphaproteobacteria | Caulobacterales | Caulobacteraceae | Caulobacter |
| **27 (>10%)** | **Proteobacteria** | **Gammaproteobacteria** | **Methylococcales** | **Methylococcaceae** | **Methylovulum** |
| 32 (5%) | Proteobacteria | Alphaproteobacteria | Rhizobiales | MNG7 | Unclassified |
| 36 (5%) | Proteobacteria | Alphaproteobacteria | Rhodospirillales | JG37-AG-20 | Unclassified |
| 40 (<5%) | Proteobacteria | Gammaproteobacteria | NKB5 | Unclassified | Unclassified |
| 42 (<5%) | Proteobacteria | Gammaproteobacteria | Legionellales | Legionellaceae | Legionella |
| 50 (5%) | Proteobacteria | Deltaproteobacteria | Myxococcales | 0319-6G20 | Unclassified |
| 54 (<5%) | Chloroflexi | Anaerolineae | Anaerolineales | Anaerolineaceae | Unclassified |
| 59 (<5%) | Proteobacteria | Gammaproteobacteria | Pseudomonadales | Pseudomonadaceae | Pseudomonas |
| 60 (5%) | Proteobacteria | Alphaproteobacteria | Rhizobiales | Phyllobacteriaceae | Aminobacter |
| 63 (<5%) | Proteobacteria | Alphaproteobacteria | Rhizobiales | Rhizobiales_Incertaw Sedis | Rhizomicrobium |
| 69 (5%) | Proteobacteria | Alphaproteobacteria | Rhizobiales | Rhizobiales_Incertaw Sedis | Bauldia |
| 70 (<5%) | Proteobacteria | Alphaproteobacteria | Rhizobiales | Methylobacteriaceae | Methylobacterium |
| 74 (<5%) | Proteobacteria | Alphaproteobacteria | Rhizobiales | Alphal cluster | Unclassified |
| 84 (5%) | Proteobacteria | Alphaproteobacteria | Rhodospirillales | Rhodospirillaceae | Ferrovibrio |
| 90 (<5%) | Proteobacteria | Gammaproteobacteria | Chromatiales | Halothiobacillaceae | Halothiobacillus |
| 93 (<5%) | Proteobacteria | Deltaproteobacteria | Myxococcales | Phaselicystidaceae | Phaselicystis |
| 94 (5%) | Proteobacteria | Gammaproteobacteria | Methylococcales | Crenotrichaceae | Crenothrix |
| 99 (<5%) | Proteobacteria | Gammaproteobacteria | Legionellales | Coxiellaceae | Aquicella |
