# Supplementary material for: Biotic Interactions in Microbial Communities as Modulators of Biogeochemical Processes: Methanotrophy as a Model System
Source: Front Microbiol. 2016 Aug 23;7:1285. doi: 10.3389/fmicb.2016.01285 (PMC4993757; doi:10.3389/fmicb.2016.01285)
Supplement: Table S3 — Co-occurring OTUs with >1% relative abundance derived from the “heavy” fraction of a 13C-CH4 labeled community in a grassland soil. Classification of OTUs is as given in Daebeler et al. (2014). Bold and gray scripts denote MOB and methylotroph, respectively. [file Table3.DOCX]

**Table S3**: Co-occurring OTUs with >1 % relative abundance derived from the ‘heavy’ fraction of a ^13^C-CH_4_ labeled community in a grassland soil. Classification of OTUs is as given in Daebeler et al. (2014). Bold and grey scripts denote MOB and methylotroph, respectively.

| OTU  (~relative abundance) | Phyla | Class | Order | Family | Genus |
| --- | --- | --- | --- | --- | --- |
| **1 (> 9%)** | **Proteobacteria** | **Gammaproteobacteria** | **Methylococcales** | **Methylococcaceae** | **Methylosarcina** |
| 5 (>3%) | Proteobacteria | Gammaproteobacteria | Pseudomonadales | Pseudomonadaceae | Pseudomonas |
| 6 (>3%) | Proteobacteria | Gammaproteobacteria | Pseudomonadales | Pseudomonadaceae | Pseudomonas |
| **15 (9%)** | **Proteobacteria** | **Alphaproteobacteria** | **Rhizobiales** | **Methylococcaceae** | **Methylosinus** |
| 17 (<3%) | Proteobacteria | Gammaproteobacteria | Enterobacteriales | Enterobacteriaceae | Raoultella |
| 41 (<3%) | Proteobacteria | Gammaproteobacteria | Xanthomonadales | Xanthomonadaceae | Pseudoxanthomonas |
| 43 (3%) | Proteobacteria | Alphaproteobacteria | Rhizobiales | Bradyrhizobiaceae | Bradyrhizobium |
| 52 (3%) | Firmicutes | Bacilli | Bacillales | Bacillaceae | Bacillus |
| 55 (<3%) | Thaumarchaeota | SAGMCG-1 | Unclassified | Unclassified | Unclassified |
| 74 (<3%) | Actinobacteria | Actinobacteria | Frankiales | Acidothermaceae | Acidothermus |
| 88 (<3%) | Chloroflexi | KZNMV-5-B42 | Unclassified | Unclassified | Unclassified |
| 89 (<3%) | Proteobacteria | Alphaproteobacteria | Rhizobiales | Methylobacteriaceae | Methylobacterium |
| 91 (<3%) | Cyanobacteria | Cyanobacteria | SubsectionIII | Familyl | Leptolyngbya |
| 122 (<3%) | Proteobacteria | Gammaproteobacteria | Acidithiobacillales | TX1A-55 | Unclassified |
| **125 (3%)** | **Proteobacteria** | **Gammaproteobacteria** | **Methylococcales** | **Methylococcaceae** | **Methylobacter** |
| 167 (3%) | Proteobacteria | Gammaproteobacteria | Xanthomonadales | Xanthomonadaceae | Rehaibacterium |
| 197 (<3%) | Chloroflexi | Anaerolineae | Anaerolineales | Anaerolineaceae | Unclassified |
| 226 (<3%) | Proteobacteria | Alphaproteobacteria | Rhodospirillales | DA111 | Unclassified |
