# Supplementary material for: Biotic Interactions in Microbial Communities as Modulators of Biogeochemical Processes: Methanotrophy as a Model System
Source: Front Microbiol. 2016 Aug 23;7:1285. doi: 10.3389/fmicb.2016.01285 (PMC4993757; doi:10.3389/fmicb.2016.01285)
Supplement: Table S4 — Co-occurring OTUs with >1% relative abundance derived from the “heavy” fraction of a 13C-CH4 labeled community in a rice paddy soil. Classification of OTUs is as given in Zheng et al. (2014). Bold and gray scripts denote MOB and methylotroph, respectively [file Table4.DOCX]

**Table S4**: Co-occurring OTUs with >1 % relative abundance derived from the ‘heavy’ fraction of a ^13^C-CH_4_ labeled community in a rice paddy soil. Classification of OTUs is as given in Zheng et al. (2014). Bold and grey scripts denote MOB and methylotroph, respectively.

| OTU  (~relative abundance) | Phyla | Class | Order | Family | Genus |
| --- | --- | --- | --- | --- | --- |
| **1 (>20%)** | **Proteobacteria** | **Gammaproteobacteria** | **Methylococcales** | **Methylococcaceae** | **Methylomicrobium** |
| **2 (>20%)** | **Proteobacteria** | **Gammaproteobacteria** | **Methylococcales** | **Methylococcaceae** | **Methylomicrobium** |
| 3 (5%) | Proteobacteria | Betaproteobacteria | TRA3-20 | Unclassified | Unclassified |
| **5 (>10%)** | **Proteobacteria** | **Alphaproteobacteria** | **Rhizobiales** | **Methylocystaceae** | **Methylocystis** |
| **7 (<10%)** | **Proteobacteria** | **Gammaproteobacteria** | **Methylococcales** | **Methylococcaceae** | **Metylobacter** |
| 11 (<5%) | Proteobacteria | Betaproteobacteria | Hydrogenophilales | Hydrogenophilaceae | Sulfuricella |
| 13 (<5%) | Proteobacteria | Gammaproteobacteria | Xanthomonadales | Xanthomonadaceae | Arenimonas |
| 20 (5%) | Chloroflexi | KD4-96 | Unclassified | Unclassified | Unclassified |
| 22 (<5%) | Proteobacteria | Gammaproteobacteria | Xanthomonadales | Xanthomonadaceae | Lysobacter |
| 24 (<5%) | Proteobacteria | Betaproteobacteria | Methylophilales | Methylophilaceae | Methylobacillus |
| 50 (<5%) | Gemmatimonadetes | Gemmatimonadetes | Gemmatimonadales | Gemmatimonadaceae | Unclassified |
| 55 (<5%) | Proteobacteria | Deltaproteobacteria | Myxococcales | Haliangiaceae | Haliangium |
| 81 (<5%) | Proteobacteria | Alphaproteobacteria | Rhizobiales | Hyphomicrobiaceae | Hyphomicrobium |
| 82 (<5%) | Proteobacteria | Betaproteobacteria | Burkholderiales | Comamonadaceae | Piscinibacter |
| 118 (<5%) | Gemmatimonadetes | Gemmatimonadetes | Gemmatimonadales | Gemmatimonadaceae | Unclassified |
| 145 (<5%) | Proteobacteria | Gammaproteobacteria | Xanthomonadales | Solimonadaceae | Unclassified |
| **207 (<10%)** | **Proteobacteria** | **Gammaproteobacteria** | **Methylococcales** | **Methylococcaceae** | **Methylomicrobium** |
| **1615 (<10%)** | **Proteobacteria** | **Gammaproteobacteria** | **Methylococcales** | **Methylococcaceae** | **Metylobacter** |
| **2641 (<10%)** | **Proteobacteria** | **Gammaproteobacteria** | **Methylococcales** | **Methylococcaceae** | **Methylomicrobium** |
