# Supplementary material for: Biotic Interactions in Microbial Communities as Modulators of Biogeochemical Processes: Methanotrophy as a Model System
Source: Front Microbiol. 2016 Aug 23;7:1285. doi: 10.3389/fmicb.2016.01285 (PMC4993757; doi:10.3389/fmicb.2016.01285)
Supplement: Table S5 — Co-occurring OTUs with >1% relative abundance derived from the “heavy” fraction of a 13C-CH4 labeled community in oilsands tailings ponds. Classification of OTUs is as given in Saidi-Mehrabad et al. (2013). Bold and gray scripts denote MOB and methanol-oxidizer, respectively. [file Table5.DOCX]

**Table S5**: Co-occurring OTUs with >1 % relative abundance derived from the ‘heavy’ fraction of a ^13^C-CH_4_ labeled community in oilsands tailings ponds. Classification of OTUs is as given in Saidi-Mehrabad et al. (2013). Bold and grey scripts denote MOB and methanol-oxidizer, respectively.

| OTU  (~relative abundance) | Phyla | Class | Order | Family | Genus |
| --- | --- | --- | --- | --- | --- |
| 2 (>15%) | Proteobacteria | Betaproteobacteria | Rhodocyclales | Rhodocyclaceae | Thauera |
| 3 (10%) | Proteobacteria | Betaproteobacteria | Burkholderiales | Comamonadaceae | Hydrogenophaga |
| 17 (<5%) | Firmicutes | Erysipelotrichia | Erysipelotrichales | Erysipelotrichaceae | Erysipelothrix |
| **32 (>10%)** | **Proteobacteria** | **Gammaproteobacteria** | **Methylococcales** | **Methylococcaceae** | **Methylocaldum** |
| 35 (<5%) | Tenericutes | Mollicutes | Acholeplasmatales | Acholeplasmataceae | Acholeplasma |
| 61 (<10%) | Proteobacteria | Alphaproteobacteria | Caulobacterales | Caulobacteraceae | Phenylobacterium |
| 71 (<5%) | Spirochaetae | Spirochaetes | Spirochaetales | Spirochaetaceae | Spirochaeta |
| 72 (5%) | Proteobacteria | Betaproteobacteria | Hydrogenophilales | Hydrogenophilaceae | Tepidiphilus |
| 87 (5%) | Proteobacteria | Gammaproteobacteria | WN-HWB-116 | Unclassified | Unclassified |
| 91 (<5%) | Proteobacteria | Gammaproteobacteria | Chromatiales | Halothiobacillaceae | Thiovirga |
| 107 (15%) | Proteobacteria | Betaproteobacteria | Burkholderiales | S3t2d-1089 | Unclassified |
| 114 (<10%) | Proteobacteria | Actinobacteria | Actinobacteria | PeM15 | Unclassified |
| 157 (15%) | Proteobacteria | Gammaproteobacteria | Thiotrichales | Thiotrichaceae | Beggiatoa |
| 182 (<5%) | Proteobacteria | Gammaproteobacteria | Xanthomonadales | Solimonadaceae | Solimonas |
| 188 (<5%) | Proteobacteria | Betaproteobacteria | Burkholderiales | Comamonadaceae | Hydrogenophaga |
